# Tobacco smoking policies in Australian alcohol and other drug treatment services, agreement between staff awareness and the written policy document

**DOI:** 10.1186/s12889-016-3968-y

**Published:** 2017-01-17

**Authors:** Eliza Skelton, Billie Bonevski, Flora Tzelepis, Anthony Shakeshaft, Ashleigh Guillaumier, Adrian Dunlop, Sam McCrabb, Kerrin Palazzi

**Affiliations:** 1 School of Medicine & Public Health, Faculty of Health & Medicine, The University of Newcastle, 1 University Drive, Callaghan, NSW 2308 Australia; 2 Hunter New England Population Health, Hunter New England Local Health District, Longworth Avenue, Wallsend, NSW 2287 Australia; 3 National Drug and Alcohol Research Centre, The University of New South Wales, 22-32 King Street, Randwick, NSW 2031 Australia; 4 Drug & Alcohol Clinical Services, Newcastle Community Health Centre, Hunter New England Local Health District, Level 3 670 Hunter Street, Suite 8, Newcastle West, NSW 2302 Australia; 5 Clinical Research Design, Information Technology and Statistical Support, Hunter Medical Research Institute, 1 Kookaburra Circuit, New Lambton Heights, NSW 2305 Australia

**Keywords:** Alcohol and other drug treatment, Policy, Smoke-free, Tobacco smoking, Enforcement

## Abstract

**Background:**

Comprehensive smoke-free policy in the alcohol and other drug (AOD) setting provides an opportunity to reduce tobacco related harms among clients and staff. This study aimed to examine within AOD services: staff awareness of their service’s smoking policy compared to the written policy document and staff and service factors associated with accurate awareness of a total ban and perceived enforcement of a total ban.

**Methods:**

An audit of written tobacco smoking policy documents and an online cross-sectional survey of staff from 31 Australian AOD services. In addition, a contact at each service was interviewed to gather service-related data.

**Results:**

Overall, 506 staff participated in the survey (response rate: 57%). Nearly half (46%) perceived their service had a total ban with 54% indicating that this policy was always enforced. Over one-third (37%) reported a partial ban with 48% indicating that this policy was always enforced. The audit of written policies revealed that 19 (61%) services had total bans, 11 (36%) had partial bans and 1 (3%) did not have a written smoking policy. Agreement between staff policy awareness and their service’s written policy was moderate (Kappa 0.48) for a total ban and fair (Kappa 0.38) for a partial ban. Age (1 year increase) of staff was associated with higher odds of correctly identifying a total ban at their service.

**Conclusions:**

Tobacco smoking within Australian AOD services is mostly regulated by a written policy document. Staff policy awareness was modest and perceived policy enforcement was poor.

## Background

Tobacco use is common in clients attending alcohol and other drug (AOD) treatment with a recent international systematic review reporting a pooled estimate of smoking prevalence of 84% in this population [1]. Smokers in AOD treatment have heavier nicotine dependence, [2] smoke more cigarettes per day and smoke for longer durations than tobacco smokers from the general population [3–5]. Consequently, clients receiving AOD treatment are likely to be more vulnerable to the negative health consequences of smoking, and are more likely to die prematurely of tobacco-related diseases [6] than of alcohol [7] or illicit drug-related causes [8].

The implementation of smoke-free policies in the AOD treatment setting is an important component of a comprehensive approach to addressing tobacco use among clients entering AOD services. Total bans, where smoking is completely prohibited [9] are more effective at reducing exposure to second-hand smoke [10], reducing cigarette consumption and increasing smoking cessation than partial bans, where smoking is permitted in designated areas or at scheduled times [10]. Factors suggested to enhance the successful implementation of tobacco smoke-free policies include strong commitment from service leadership [11, 12], active promotion of the written policy document [12, 13], staff participation, extensive training [12] and consistent enforcement of the written policy [14].

Few studies have examined the type and content of smoking policies in AOD treatment settings. A US study of 13,094 AOD treatment services found that just over one-third (35%) had a total ban smoking policy. Another US study, examining 408 outpatient methadone maintenance program clinic leaders found that the majority reported having a written smoking policy for staff (82%) and clients (73%) [15]. Most banned indoor smoking for both staff (94.8%) and clients (95.8%) however were less restrictive with outdoor bans for staff (55.2%) and clients (47.8%) [15]. A Canadian survey of 125 residential rehabilitation services revealed that over half of all services banned indoor smoking (52.8%) [16]. A survey of 417 staff from 260 Australian services found that most managers (82.5%) and staff (76.7%) reported that their service had a tobacco smoking policy [17]. The 2005 study found that less than 10% of managers (9.9%) and other staff (9.3%) indicated that their service had a total smoking ban [17]. The majority of managers (80.9%) and other staff (72.5%) indicated that their total smoking ban or partial ban prohibiting inside smoking was always enforced [17].

Notably, no prior research has directly compared staff self-reported awareness of their AOD services’ smoke-free policy with the written policy document nor examined service and staff characteristics associated with the accurate awareness of a total ban smoking policy. This study aims to examine:

1. staff awareness of tobacco smoking policy and enforcement within their service
2. the written tobacco smoking policies (total ban, partial ban, no/unrestricted) in AOD treatment services
3. staff awareness of their service’s smoking policy compared to the written policy document
4. staff and service factors associated with accurate awareness of a total smoking ban policy
5. staff and service factors associated with perceived enforcement of a total smoking ban policy

## Methods

### Study design

An online cross-sectional survey was conducted with staff from 31 AOD treatment services in July-October 2014. In addition, a site contact at each service was interviewed to gather service-related data for the same period and to obtain a copy of the treatment services’ written smoking policy.

### Setting

Thirty-one AOD services participating in four states and territories of Australia: New South Wales (NSW), Australian Capital Territory (ACT), Queensland (Qld) and South Australia (SA) were included in this survey. Government services were recruited through key contacts such as directors of health services while non-government services were invited through peak bodies who promoted the research in their newsletters. Eligible services were those with face to face contact with at least 50 clients per year (that is, larger services). Of 32 eligible services that expressed interest in participating, 31 completed the study.

### Sample

Eligible participants were current voluntary, casual, part-time or full-time members of staff at participating services.

### Procedure

The research team sent an invitation email containing the participant information letter and the hyperlink to the online survey to a study contact at each site for distribution to all staff. Survey participation constituted consent. Three reminder emails were sent at weekly intervals to all staff members. At the end of the 4-week survey period, services that had a written tobacco smoking policy were asked to email a copy to the research team. Ethics approval was obtained through the Hunter New England Human Research Ethics Committee (HREC), ACT Health HREC, SA Health HREC and the University of Newcastle HREC.

### Measures

#### Staff online survey

Survey items were based on existing questionnaires from similar studies [17, 18].

##### AOD treatment staff characteristics

Staff reported their gender, age, work role, number of years employed in the AOD field, number of years employed at the service, smoking status. Staff also reported on the substances commonly addressed by their service. Respondents could select as many responses as applied from a list of substances (any psychotic drug, alcohol, benzodiazepines and other sedatives, cannabis, heroin and other opioids, psychostimulants incl. amphetamines, any other illegal drug).

##### Staff awareness of tobacco smoking policy

Staff were asked “Does your service have a written policy stating a total ban on smoking? (Yes, No, Unsure).” Staff who reported no or unsure were then asked “Does your service have a written policy stating any restrictions on smoking? (Yes, No, Unsure)”. Staff reporting yes to a written policy stating restrictions on smoking were asked whether smoking was permitted: inside the service, outside the service, in an indoor designated area, in an outdoor designated area, in service cars, on home visits (staff only). These questions were asked in relation to staff and clients separately. Staff were also asked “Has the service’s smoking policy changed in the last 12 months? (Yes, No)”.

##### Enforcement of tobacco smoking policy

Staff reporting that their organisation had a total smoking ban or partial smoking ban were asked to rate on a 6 point Likert scale (always, often, sometimes, rarely, never, unsure)“How often is the total/partial ban enforced at your service?”.

#### Site contact telephone interview

##### AOD treatment service characteristics

At the time of the online survey, a member of the research team called the site contact and verbally collected information on the following: government-managed or non-government managed service, AOD treatment program (residential rehabilitation/therapeutic community, opiate treatment/methadone and buprenorphine maintenance, out-patient counselling, specialist withdrawal unit, other-harm minimization and other-area health) and location (major city, inner/outer regional area) based on the Accessibility/Remoteness Index of Australia (ARIA+) [19, 20].

#### Audit of written tobacco smoking policy documents

Policy documents were reviewed and rated using a coding tool developed by the research team. Policies were categorized into one of three types: 1) total smoking ban (smoking is prohibited in all indoor areas, the grounds of the service and within vehicles owned or leased by the service); 2) partial smoking ban (smoking is restricted to designated areas, scheduled times within the day or by an exemption pass held by the individual); and 3) unrestricted/no smoking ban policy (smoking is permitted within indoor and outdoor areas at the service/no formal smoking document). Policy documents classified as a partial smoking ban were then further examined to determine in which of the following areas tobacco smoking was permitted for clients/visitors and for staff: inside the service; outside the service; in an indoor designated area; in an outdoor designated area; in service’s cars; on home visits (staff only). Each of the areas were coded as 1 = yes, 2 = no or 3 = unspecified/missing.

#### Statistical analysis

Descriptive statistics of staff and service characteristics are presented by frequencies and percentages for categorical variables and means (standard deviation) or median (interquartile range IQR; Quartile1- 3) for continuous variables depending on the distribution. Agreement between staff awareness of their service’s smoking policy and the written policy document was examined using Cohen’s Kappa statistic (95% CI, *p*-value) and percent perfect agreement with interpretations of Kappa statistics and the strength of agreement as per Landis and Koch: <0.00 = Poor; 0.00–0.20 = Slight; 0.21–0.40 = Fair; 0.41–0.60 = Moderate; 0.61–0.80 = Substantial; 0.81–1.00 = Almost Perfect [21].

Binary logistic regression was used to examine the association between staff and service characteristics and the accurate identification of the presence of a total smoking ban and the association between staff and service characteristics and perceived enforcement of a total smoking ban. Variables included in the logistic regression were selected *a priori*. Collinearity of variables was checked using variance inflation factors (VIFs). Correlation within individuals from the same service was examined by fitting a model general estimating equation (GEE) with and without a repeated statement for each service and examining model fit. If the model fit was not found to be improved (reduction of Quasi- information criterion [QIC] of more than 4 points) then logistic regression without clustering was used. Adjusted odds ratios with 95% confidence intervals and *p*-values were calculated. Significance was determined at *p* < 0.05. SAS 9.4 (SAS Institute Inc., Cary, NC, USA) was used for all analyses.

## Results

### AOD treatment service characteristics

The majority of services were: located within a major city (77%), government-managed (58%) and residential rehabilitation services (42%) or out-patient counselling services (29%).

### AOD staff characteristics

Of 882 AOD managers and staff invited, 506 participated in the survey, giving an overall response rate of 57.4%. The majority of survey respondents were female (70%) and had a mean age of 45 years (SD = 12). Overall 16% were in a management role (CEO, manager, director, coordinator and/or team leader; see Table 1). Most staff indicated that they had current client contact (76%) and the substances commonly addressed by their service were: alcohol (82%), heroin and other opioids (82%) and cannabis (79%). In terms of current smoking status, 43% were ex-smokers, 32% were never smokers and 25% were daily or occasional smokers.

**Table 1:** AOD staff characteristics

Characteristic	Number^a^	Percent
Gender		
Female	322	70
Male	138	30
Age in years (mean, SD)	45 (12)	
Role		
Manager	81	16
Nurse	126	25
Caseworker	91	18
Counsellor	57	11
Administration	48	9.6
Psychologist	20	4
Social worker	18	3.6
Medical Practitioner (specialist/generalist)	15	3
Health Educator	14	2.8
Researcher	7	1.4
Volunteer	4	0.8
Pharmacist	2	0.4
Other	13	2.6
Smoking status		
Ex-smoker	188	43
Non-smoker	142	32
Daily/Occasional smoker	108	25
Employment status		
Full-time	307	62
Part-time	155	32
Casual	26	5.3
Volunteer	3	0.6
Highest work qualification		
School certificate/Higher school certificate	18	5
TAFE^b^ certificate/diploma	118	32
University undergraduate/postgraduate degree	233	63
Number of years at organisation		
< 1 year	62	13
1–3 years	127	26
4–6 years	106	22
7–9 years	66	14
≥ 10 years	127	26
Number of years in the AOD field		
< 1 year	39	8
1–3 years	101	21
4–6 years	88	18
7–9 years	66	14
≥ 10 years	193	40
Substances addressed by service		
Heroin and other opioids	405	82
Alcohol	405	82
Cannabis	388	79
Psychostimulants incl. Amphetamines	386	78
Benzodiazepines incl. Other Sedatives	332	67
Any other illegal drug	274	56
Any other injectable drug	261	53
New and emerging substances incl. stimulant-like	241	49
substance, cannabis-like substance, hallucinogen-like		
substance		

^a^May not equal 506 for staff due to missing data

^b^TAFE: Technical and Further Education

### Staff awareness of tobacco smoking policy

Almost half of staff (46%, *n* = 210) reported working in a service with a written total smoking ban policy, 37% (*n* = 167) reported a written partial smoking ban, 12% (*n* = 55) reported they were unsure and 5% (*n* = 23) reported unrestricted smoking. Staff reporting that their service had a written partial smoking ban identified that client and staff smoking was permitted in the following areas: outdoor designated area (client 78%, staff 71%), outside the service (clients 77%, staff 74%), inside the service (clients 14%, staff 13%), indoor designated area (clients and staff 1%), in service cars (clients 0%, staff 1%). The majority of respondents noted that their service’s smoking policy had not changed in the last 12 months (42%).

### Enforcement of tobacco smoking policy

Of all staff reporting that their service had a written total smoking ban, 54% stated it was always enforced. Of those who correctly identified their services’ total ban policy, 55% stated that this ban was always enforced. Of all staff reporting that their services had a written partial smoking ban, 48% stated that it was always enforced. Of those staff who correctly identified their services’ partial smoking ban policy 57% reported that this ban was always enforced.

### Audit of written tobacco smoking policy documents

The audit revealed that 61.3% (*n* = 19) of services had a total smoking ban, 35.5% (*n* = 11) had a partial smoking ban and 3.2% (*n* = 1) did not have a written tobacco smoking policy document. For services with partial smoking bans, the areas where smoking was permitted were: outdoor smoking area (clients 100%, staff 91%), outside the service (clients 100%, staff 91%), inside the service (clients 0%, staff 0%), indoor smoking area (clients 0%, staff 0%), inside service vehicle (clients 0%, staff 0%), on home visit (staff only 0%).

### Agreement between staff awareness of type of smoking ban policy and the written total, partial or no smoking ban policy

The majority of respondents (64%) from a service with a total smoking ban correctly identified that their service had this form of policy, the kappa value was 0.48 (95% CI 0.41, 0.55, *p* = 0.04; 73.3% perfect agreement; see Table 2) indicating moderate agreement. Just over two-thirds (67.2%) of respondents from services with a written partial smoking ban policy correctly identified their services’ written policy document, the kappa value was 0.38 (95% CI 0.29, 0.47, *p* < 0.001; 72.5% perfect agreement) indicating fair agreement. Staff who reported that their services did not have a total smoking ban or partial smoking ban policy were considered as reporting that their service had no smoking ban policy document. Only 25% of respondents from the service with no written tobacco smoking policy correctly reported that they did not have a smoking ban. The kappa value was 0.02 (−0.04, 0.08; *p* = 0.07; 82.2% perfect agreement) indicating slight agreement.

**Table 2:** Agreement between staff awareness of the type of smoking ban policy and written policy document

Awareness	Written policy		% Perfect agreement	Kappa^a^ (95% CI)	*P*-value
	No	Yes			
Total ban			73.3%	0.48 (0.41,0.55)	*0.04*
No/Unsure	128 (96.2%)	115 (36.4%)			
Yes	5 (3.8%)	201 (63.6%)			
Partial ban			72.5%	0.38 (0.29,0.47)	*<0.001*
No/Unsure	241 (74.6%)	41 (32.8%)			
Yes	82 (25.4%)	84 (67.2%)			
No ban			82.2%	0.02 (−0.04, 0.08)	*0.07*
No	367 (83.2%)	6 (75%)			
Yes	74 (16.8%)	2 (25%)			

^a^<0 Poor; 0–0.2 Slight; 0.21-0.4 Fair; 0.41-0.6 Moderate; 0.61-0.8 Substantial; 0.81-1 Almost Perfect

### Agreement between staff awareness of designated smoking areas and those in written partial ban policy documents

For those who had a partial smoking ban, percent perfect agreement between staff awareness of designated smoking areas and the staff areas identified in the written policy was good overall (see Table 3). No kappa estimates were available for the following areas: inside the service, inside organisation vehicles, on home visits as all services prohibited smoking. There were also found to be no indoor designated smoking areas. Therefore, kappa was only available for staff smoking areas outside of the service and outdoor smoking areas, indicating poor agreement (Kappa = −0.0014 and −0.06 respectively).

**Table 3:** Staff awareness of designated smoking areas and areas in written partial ban smoking policy documents

	Client smoking areas			Staff smoking areas		
Location	% Perfect agreement	Kappa (95% CI)^a^	*P* value	% Perfect agreement	Kappa (95% CI)^a^	*P* value
Inside the service^b^	79.2%			80.8%		
Outside the service	78.4%			72.8%	−0.0014 (−0.1, 0.1)	0.98
Indoor smoking area^b^	97.6%			99.2%		
Outdoor smoking area	84.1%			72.2%	−0.06 (−0.11, −0.06)	0.25
Inside service vehicle^b^	100%			100%		
On home visit^b^				98.5%		

^a^<0 Poor; 0–0.2 Slight; 0.21-0.4 Fair; 0.41-0.6 Moderate; 0.61-0.8 Substantial; 0.81-1 Almost Perfect

^b^No kappa estimate was available as all services with a written partial ban policy documented that smoking was not permitted: inside the organisation, inside service vehicles and on home visit. No services had a designated indoor smoking area. Therefore, a kappa statistic was unable to be calculated as this requires a 2×2 cross-tabulation

### Staff and service characteristics associated with accurate awareness of a total smoking ban policy

Table 4 presents the results of the logistic regression. Increasing age (per year) of respondents was associated with having higher odds (OR 1.03, 95% CI 1.00–1.06, *p* = 0.02) of accurately reporting their services’ total smoking ban policy.

**Table 4:** Characteristics associated with accurate awareness of a total smoking ban policy (*n* = 191)

	Unadjusted		Adjusted	
Characteristic	OR (95% CIs)	*p*-value	OR (95% CIs)	*p*-value
Government organisation (reference: non- government organisation)	1.47 (0.88–2.47)	0.14	1.14 (0.62–2.08)	0.68
Female (reference: Male)	0.68 (0.40–1.15)	0.15	0.76 (0.43–1.34)	0.34
Age	1.05 (1.03–1.07)	0.00	1.03 (1.00–1.06)	*0.02*
Manager (reference: Staff)	2.06 (0.98–4.36)	0.06	1.47 (0.64–3.38)	0.36
Smoking Status (reference: Current smoker)	.	0.00	.	0.06
Ex-smoker	2.71 (1.45–5.08)	0.00	2.18 (1.12–4.25)	0.02
Non-smoker	1.43 (0.76–2.69)	0.27	1.33 (0.69–2.57)	0.40
Number of years in the AOD field (reference: 10+ years)	.	0.00	.	0.77
less than 1 year	0.34 (0.13–0.88)	0.03	0.62 (0.21–1.87)	0.40
1–3 years	0.34 (0.18–0.63)	0.00	0.69 (0.31–1.55)	0.37
4–6 years	0.52 (0.26–1.03)	0.06	0.80 (0.36–1.80)	0.59
7–9 years	0.45 (0.21–0.94)	0.03	0.61 (0.27–1.39)	0.24

### Staff and service characteristics associated with perceived enforcement of a total smoking ban policy among staff with accurate awareness of a total smoking ban policy (*n* = 106)

None of the factors examined were found to be associated with perceived enforcement of a total smoking ban.

## Discussion

The majority of Australian AOD treatment staff surveyed in this study identified that tobacco smoking at their service was regulated by a written policy restricting tobacco smoking. Approximately half stated that the policy at their service was always enforced. Where staff reported partial smoking restrictions, smoking was often permitted in outdoor designated areas. The audit of written policy documents revealed that tobacco smoking is largely regulated by either a total or partial ban with only one service not found to have a formal written policy document. No service with a written policy outlining partial restrictions permitted the use of tobacco smoking indoors; however smoking was permitted in outdoor areas in 10 out of 11 services. Staff were found to have a moderate awareness of their tobacco smoking policy document with the majority correctly identifying whether their service had a total, partial or no/unrestricted tobacco smoking policy. Age of respondent was significantly associated with accurate awareness of a total smoking ban while no factors were associated with higher odds of correctly identifying a total ban at their service.

Compared to international studies examining smoke-free policy within the AOD treatment setting [15, 16, 22] our findings suggest that total ban smoking policies appear to be more prevalent in Australian AOD services. A 2015 US study of 13,094 AOD services found that 35% had implemented total ban smoking policies [22] while a 2003 Canadian study of 125 AOD services found that 53% had total ban smoking policies [16]. The only other Australian study on AOD treatment smoking policies was conducted over 15 years ago and in comparison found relatively few services with a total smoking ban (managers 9.9%, staff 9.3%) [17]. The increased adoption of total ban smoking policies is not surprising given the sustained national tobacco control measures (excise increases, bans on point of sale of tobacco product advertisement, plain packaging, further regulations to the smoke-free act) denormalising tobacco smoking over the past decade [23].

This study revealed that accuracy in policy awareness was associated with increasing age of treatment staff. In the broader AOD treatment staff literature, knowledge of tobacco smoking and smoking cessation care (SCC) strategies appears to be influenced by the amount of continuing education in nicotine dependence [24]. It is possible that older treatment staff have been provided with more smoking cessation training throughout their careers. It may also be likely that they have been present for updates and revisions to their service’s tobacco smoking policy therefore through this exposure are more likely to accurately recall the written document.

While our study did not find smoking status overall to be significantly associated with accurate policy awareness, treatment staff who identified as ex-smokers were more likely to accurately report their service’s policy compared to current smokers. Studies examining AOD treatment staff smoking status report that individuals identifying as non-smokers and ex-smokers have greater support of a total ban smoking policy [25]. It may be that these individuals are more supportive of tobacco control and therefore more likely to be able to accurately identify their policy document.

### Implications

The results of this study indicate that although AOD treatment services have written total and partial smoking bans, a large proportion of treatment staff are not aware of the type and content contained within their service’s document. Staff awareness could be improved through a number of strategies including, but not limited to, policy promotion, training and smoking cessation treatment protocols. It is essential that policy promotion involve all levels of staff to allow for buy-in and ownership [26, 27]. This level of engagement by staff is critical for the sustainability and enforcement of a total ban smoking policy [28]. Policy promotion should also involve the development of smoke-free signage that is strategically placed in areas in which smoking occurs but is not permitted. Further, information should be provided on the rights and responsibilities of staff in ensuring policy compliance along with the monitoring and reporting protocols for incidents of non-compliance. To ensure compliance, enforcement measures such as fines and inspections, will need to be developed by the service. The type and perceived success of current enforcement measures of services with a total or partial ban were not explored in this study, further examination is therefore required.

Smoking cessation specific training should be provided to all members of staff. Considering the significant association between age of AOD staff and accuracy of policy, opportunities for further training for younger members of staff, particularly those who are new to the AOD field should also be provided. To support smoke-free policy, services need to develop protocols specific to the assessment and treatment of tobacco smoking for clients and staff. These processes should be implemented as part of usual care practice at the service.

Partial bans continue to permit tobacco smoking, furthering the acceptance of this practice within the AOD treatment setting. Evidence suggests that exposure to second hand smoke remains high in services with partial bans and are less sustainable than total ban smoking policies [9, 29]. AOD services with no written policy or partial bans should set goals to work towards the implementation of a total ban at their service. Importantly, the implementation of a total ban smoking policy is both feasible and acceptable for AOD services and their clients [30–32]. The adoption of a total ban smoking policy has the potential to create culture change within the service and has implications for clinical practice [33]. Staff attitudes are found to become more favourable towards addressing client smoking in addition to facilitating the assessment and delivery of consistent SCC [10, 22, 34].

### Strengths and limitations

This study is the first to examine agreement between what staff identify their AOD service’s smoking policy to be with what is documented in the written policy. As the results show, reliance on staff self-report of smoking policies can be inaccurate. This is also the first study to examine both staff and service factors associated with staff correctly identifying their smoking policy and enforcement of such a ban. Other study strengths include a large sample of AOD staff recruited from a range of AOD services across a number of Australian states and territories. The response rate is comparable to similar studies conducted with AOD staff [17, 18, 35]. However the generalizability of the study findings may be limited as the services that volunteered for the study may have been more interested and motivated in regards to tobacco control compared to the services that did not participate.

## Conclusions

Smoking policies play a significant role in reducing exposure to second-hand smoke and creating a culture that is supportive of smoking cessation [26]. For instance, the introduction of smoke-free policies in the psychiatric inpatient setting has been associated with individual behaviour change such as increased quit attempts [10]. Although smoke-free policy is vital to ensuring change to the culture and norms of AOD services its value will be limited if awareness and enforcement of the policy is poor. The incongruity between the written policy document and staff awareness of the content and perceived enforcement in our study suggests the need for additional initiatives. Initiatives that engage all levels of staff, particularly younger members, and involve the promotion of the written policy document, the provision of staff education, the development of enforcement measures and the delivery of SCC to improve awareness and facilitate compliance are required.

The incongruity between the written policy document, staff awareness of the type of policy and enforcement is particularly concerning in the AOD setting because of the high smoking rates (84%) [1], heavier nicotine dependence [2] and the increased risk of tobacco-related diseases and mortality in AOD clients [6]. Partial enforcement of AOD services smoke-free policies would undermine the efforts to assist AOD clients to quit smoking and reduce chronic diseases in this population.

While past studies present AOD services as having a culture permissive of tobacco smoking [5], the amounting evidence for AOD services to become smoke-free and adopt smoke-free policies are steadily transitioning the setting to be supportive of smoking cessation. Overall and as exemplified in our findings, AOD services have made significant progress in implementing smoke-free policy and creating an environment that supports client cessation [26]. Despite this, improvements in policy content awareness and enforcement continues to be required. Limited staff awareness and enforcement of smoking policies in the AOD setting may convey to the workforce, the sector and clients that addressing tobacco is not a priority and that smoking is acceptable in this setting. Therefore, considerable efforts lie ahead to ensure AOD services continue to create a culture of compliance.

## Abbreviations

AOD: Alcohol and other drug
ARIA: Accessibility/Remoteness Index of Australia
SCC: Smoking cessation care
